# Mass Spectrometry Based Lipidomics: An Overview of Technological Platforms

**DOI:** 10.3390/metabo2010019

**Published:** 2012-01-05

**Authors:** Harald C. Köfeler, Alexander Fauland, Gerald N. Rechberger, Martin Trötzmüller

**Affiliations:** 1 Core Facility for Mass Spectrometry, Medical University of Graz, Stiftingtalstrasse 24, 8010 Graz, Austria; Email: martin.troetzmueller@medunigraz.at; 2 Institute of Analytical Chemistry and Food Chemistry, Graz University of Technology, Stremayrgasse 9/II, 8010 Graz, Austria; Email: alexander.fauland@tugraz.at; 3 Department for Molecular Biosciences, University of Graz, Humboldtstrasse 50/II, 8010 Graz, Austria; Email: gerald.rechberger@uni-graz.at

**Keywords:** lipidomics, mass spectrometry, LC-MS, shotgun, ESI, MALDI-TOF

## Abstract

One decade after the genomic and the proteomic life science revolution, new ‘omics’ fields are emerging. The metabolome encompasses the entity of small molecules—Most often end products of a catalytic process regulated by genes and proteins—with the lipidome being its fat soluble subdivision. Within recent years, lipids are more and more regarded not only as energy storage compounds but also as interactive players in various cellular regulation cycles and thus attain rising interest in the bio-medical community. The field of lipidomics is, on one hand, fuelled by analytical technology advances, particularly mass spectrometry and chromatography, but on the other hand new biological questions also drive analytical technology developments. Compared to fairly standardized genomic or proteomic high-throughput protocols, the high degree of molecular heterogeneity adds a special analytical challenge to lipidomic analysis. In this review, we will take a closer look at various mass spectrometric platforms for lipidomic analysis. We will focus on the advantages and limitations of various experimental setups like ‘shotgun lipidomics’, liquid chromatography—Mass spectrometry (LC-MS) and matrix assisted laser desorption ionization-time of flight (MALDI-TOF) based approaches. We will also examine available software packages for data analysis, which nowadays is in fact the rate limiting step for most ‘omics’ workflows.

## 1. Introduction

Biological systems, such as cells, comprise thousands of individual molecular lipid species, called the lipidome of an organism, which can be classified into eight major lipid categories and dozens of lipid classes and subclasses [1]. Generally, lipids fulfil three major tasks in cellular systems: Energy storage, structural functions and cellular signaling [2,3]. Within the last decade, it became increasingly evident that lipids are not only energy storing bystanders in cellular processes but are a vital part of cellular regulation processes by themselves. As their biological properties strongly depend upon their chemical structure, each molecular lipid species has an individual role in a living system. An imbalance in this system can lead to various pathophysiological conditions such as diabetes, atherosclerosis, liver steatosis, chronic inflammation and also neurodegenerative diseases to name just a few [4,5].

Improved lipidomic technologies greatly enhance the knowledge about lipid functions at the level of individual species [6]. Thin layer chromatography (TLC), the classical standard in lipid analysis, is cheap and fast, but it is very limited when it comes to identification issues below the level of lipid classes. Due to its sensitivity and selectivity mass spectrometry (MS) is the method of choice for qualitative and quantitative lipidomic analysis. Although it is not yet possible to detect and quantify all individual lipids in a given cellular system, the aim of lipidomic analysis is to determine as many individual lipids as possible.

Compared to biopolymers such as DNA, RNA, carbohydrates or proteins, lipids show much less standardized fragment mass spectra. Each lipid class has its own rules for fragmentation and its specific ionization efficiency [7], which makes development of standardized ‘all inclusive’ methods a daunting challenge. Depending on the instrumental setup, different layers of information about the molecular structure are to be discovered. Survey approaches sometimes only determine the number of fatty acyl carbons and the number of fatty acyl double bonds, whereas more focused in-depth methods are able to determine structural details down to fatty acid double bond position. Due to the diversity in molecular structures there is no single mass spectrometric approach which could cover detection of the whole lipidome of an organism, but usually it is rather a combination of different experimental platforms. The following article will focus on mass spectrometry instrumentation using electrospray ionization (ESI), atmospheric pressure chemical ionization (APCI) and MALDI, because these are nowadays the ionization techniques of choice for complex lipids with a molecular weight above 500 Da.

## 2. Direct Infusion

The first ones to propose lipidomic analysis by ESI were Han and Gross in 1994 [8]. This concept utilized the emerging combination of ESI with a triple quadrupole analyzer. Sample injection was done by a syringe pump. A few years later, the same instrumentation was successfully used for lipid analysis in combination with the first prototypes of a static nano ESI source [9] without syringe pump. The nano ESI source with a flow rate of about 80 nL/min resulted in higher ionization efficiencies than a syringe pump running at several µL/min. Although this method now widely termed ‘shotgun lipidomics’ has improved a lot in the last 15 years, the basic concept behind it remains the same. It is based on precursor ion and constant neutral loss scans of readily ionizable phospholipid headgroups, resulting in lipid class specific fragments [10]. Shotgun lipidomics avoids difficulties with concentration alterations and chromatographic abnormalities. Another advantage compared to LC-MS is the longer time which can be spent on each lipid class specific scan type. Addition of one internal standard per lipid class was shown to be sufficient for quantitation [11], because ionization of lipids is largely dependent on the class specific head group and not so much on the fatty acyl chains [12]. As there are also contradictory reports about the influence of fatty acyl chain length and unsaturation on ionization efficiency [9,13], it is advisable to extensively evaluate each individual system on this issue. One drawback of shotgun lipidomics are isobaric overlaps of the M + 2 isotope with the monoisotopic peak of the compound with one double bond less. This can be overcome by deisotoping algorithms [14]. The output format of data usually indicates the sum of fatty acyl carbons and the sum of double bonds, but not the individual composition of fatty acids.

Multi-dimensional mass spectrometry based shotgun lipidomics (MDMS-SL) is a further development of shotgun lipidomics taking into account the concept of building blocks in lipid structures [15]. MDMS-SL takes advantage of differential intrasource separation properties with various additives like Li^+^, NH_4_^+^ or Na^+^, and unique fragments for each lipid class. Glycerolipids without class specific fragments are detected by constant neutral losses of fatty acids and information about the intact lipid is drawn from the combinatorial possibilities of all monitored fatty acid neutral losses [16]. Coupled with the Nanomate® system (Advion Biosciences, Ithaca, NY), the MDMS-SL concept proves to be a powerful high throughput device because of its high degree of automatization and the enhanced sensitivity provided by a nano-ESI source. The MDMS-SL system covers quantitative analysis of various classes of glycerophospholipids, sphingolipids and glycerolipids [10].

Flow injection lipidomic analysis, a variation of shotgun lipidomics is proposed by the group of Liebisch [17]. In contrast to classical shotgun lipidomic methods, this experimental setup utilizes an HPLC apparatus coupled to a triple quadrupole analyzer. The HPLC pump runs at microflow rates and the autosampler injects samples automatically into the flow, which delivers the sample directly into the ESI source without HPLC column. The HPLC autosampler offers a higher degree of automatization than a syringe pump. Furthermore, it results in a short and concentrated sample pulse for about one minute, which can be used for data acquisition with precursor ion and constant neutral loss scans. Depending on the number of scans necessary, multiple injections per sample are possible. Quantitation is achieved by a standard addition method with multiple standard curves, featuring one internal standard and sets of lipids with different fatty acyl chain lengths and degrees of unsaturation [18]. The method is very robust, highly automated and was applied on various subclasses of glycerophospholipids, sphingolipids and sterols [18,19,20,21]. As for all low resolution direct infusion technologies it runs into its limits when isobaric nominal mass compounds derived from the same phospholipid subclass occur, like, e.g., diacyl and acyl-alkyl glycerophospholipids.

Another direct infusion approach encompasses coupling of a syringe pump and a triple quadrupole analyzer in multiple reaction monitoring (MRM) mode [22]. In this experimental setup, the anticipated precursor and product ions must be known. It allows a quick and reliable quantitation of major lipid components in a given lipid extract. On the downside, this method has a limited capability for detection of unexpected lipid species and is particularly vulnerable for overlapping isobaric compounds.

In contrast to low resolution instruments, high resolution mass spectrometers deliver accurate mass and elemental composition of ions with very high confidence. Most lipid classes have an unambiguous fingerprint due to a certain and invariable number of the heteroatoms N, O, P and S. Due to this fact, the elemental composition of precursor and often also product ions contains highly valuable information about the lipid class. The Multiple Precursor Ion Scans (MPIS) method developed on a quadrupole-TOF instrument by the group of Shevchenko [23,24] combines high resolution precursor ion scans on glycerophospholipid headgroups and fatty acyl moieties, resulting in the individual fatty acyl composition of glycerophospholipid species. The high resolving power is particularly helpful in the case of ambiguous product ions with mass differences in the first or second digit. The MPIS concept was successfully applied for quantitative global lipidome analysis in various cell systems including glycerophospholipids, glycerolipids, sphingolipids, sterols and various glycolipids [25,26,27]. The method has its limitations when one lipid class like triacylglycerol (TG) is present in bulk amounts and possibly suppresses ionization of other minor lipid classes [28].

**Figure 1:**
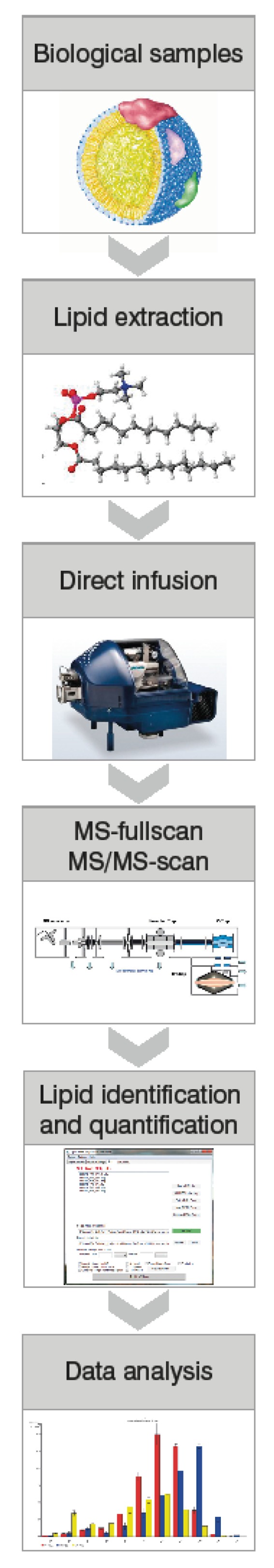
Schematic outline of a high throughput shotgun lipidomics platform consisting of an LTQ-Orbitrap mass spectrometer coupled to a NanoMate.

A further development of the MPIS concept is shotgun lipidomics with a hybrid LTQ-Orbitrap instrument coupled to the Nanomate® ion source [29]. A schematic workflow of this platform is shown in Figure 1. This system relies on accurate mass down to sub ppm range for both, precursor and product ions. With the recent development of higher energy collision-induced dissociation (HCD) even the low mass cut off problem of product ion spectra acquired in the LTQ could be overcome [30]. The Nanomate® provides plenty of time to be spent on each sample with only a few microliters of it consumed. This opens up the avenue for data-dependent acquisition of product ion spectra on all possible precursor ions, resulting in full scan precursor spectra and product ion spectra of literally every detectable lipid species at a resolution of 100,000 or more. Additionally, exact assignment of fatty acyl side chains can be achieved on a regular basis with this system. Quantitation is done by one internal standard per lipid class [31], which is sufficient to compensate for varying ionization efficiencies.

An interesting alternative to gas chromatography-mass spectrometry (GC-MS) analysis of fatty acids is published by the Welti group [32]. The CID-TOF system uses a quadrupole-TOF analyzer coupled to negative ESI direct infusion. Thereby mass selection in Q1 is turned off, Q2 fragments all ions and the TOF analyzer records intact fatty acid carboxylates with accurate mass. This provides the fingerprint of fatty acids including modified fatty acids without any prior derivatization step being necessary. Mentionable, this method only works for lipids which generate negative ions in ESI, but nevertheless comparison with GC-flame ionization detector (GC-FID) data shows good correlation [32].

## 3. LC-MS

### 3.1. Low Resolution Mass Spectrometry

**Figure 2:**
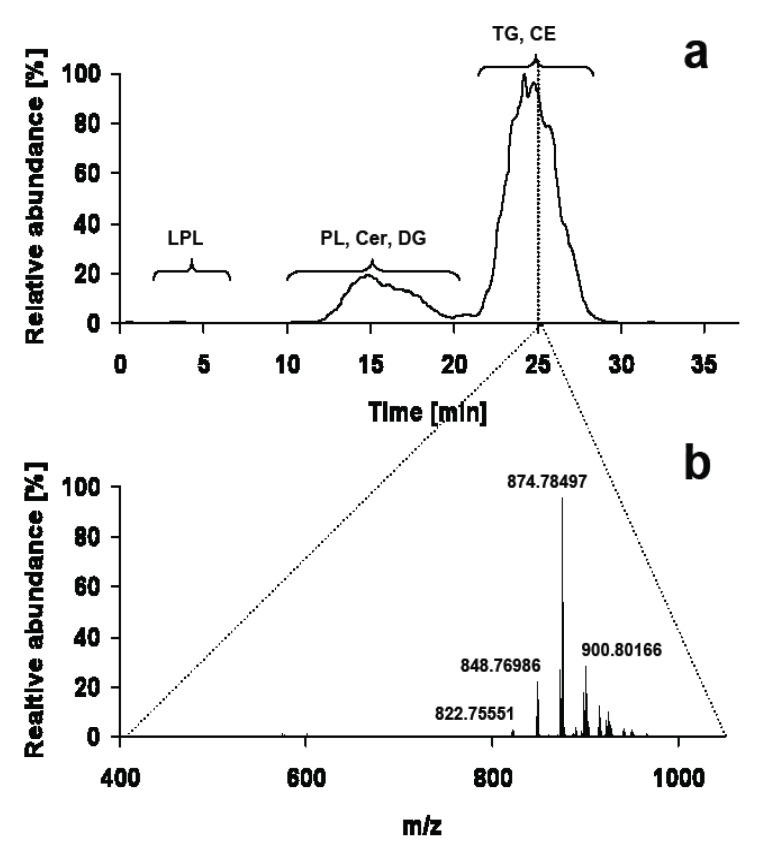
(**a**) Total ion chromatogram of a lipid droplet extract acquired on C-18 reversed phase HPLC coupled to an LTQ-FT in positive ESI mode (**b**) consisting of 487 individual full scan mass spectra at a resolution of 200,000. The expected *m/z* ranges for TG and CE are *m/z* 800–1,000 and *m/z* 600–700 respectively. Solvent A is water and solvent B acetonitrile / 2-propanol 5:2 (v/v). Both solvents contain 10 mM ammonium acetate and 0.1% HCOOH. The binary gradient starts at 50% B, increases to 70% B in 4 min and then to 100% B in another 21 min, where solvent B is held for 9 min.

The invention of ESI enabled coupling of HPLC with mass spectrometry in a highly efficient manner for the first time [33]. This instrumental combination opened up completely new analytical perspectives in lipid research by combining the separation power of HPLC with the selectivity of mass spectrometry. Complex lipid classes like glycerolipids, glycerophospholipids or even glycolipids were analytically amenable on a regular basis by chromatography coupled to mass spectrometry, now termed LC-MS. Compared to direct infusion systems HPLC adds retention time as another layer of selectivity. On one hand this results in increased specificity for lipid identification, but on the other hand it complicates quantitation, because every spectrum in an LC-MS run has to be regarded as a single event with unique matrix effects and solvent composition (Figure 2). Therefore quantitative aspects are generally more difficult to be standardized than for direct infusion methods.

Excellent examples of lipidomic LC-MS analysis have recently been shown for human plasma [34] and subcellular organelle lipidomics of TLR-4 activated macrophages [35] by the LIPID MAPS consortium. These publications show very well that the whole lipidome of an organism or tissue cannot be determined by a single experimental setup but rather by a combination of various methods, most of them LC-MS based.

The fastest and simplest way to monitor changes in a lipid profile by LC-MS is LC/single ion monitoring (SIM)/MS [36]. This method is based on ESI full scan quadrupole MS of intact molecular adduct ions. Plotting of retention time versus *m/z* and intensity provides a 3D lipidomic fingerprint of a sample, which can be used to monitor changes between statistical groups by differential profiles in a fast and comprehensive manner.

A very efficient way to maximize sensitivity is targeted lipidomics with HPLC-triple quadrupole instrumentation in MRM mode. Due to the instrument’s high dynamic range and the selectivity of retention time in conjunction with known precursor / product ion pairs, it is the method of choice for lipid quantitation. Recent developments in scan speed of triple quadrupole mass spectrometers result in a duty cycle of up to 100 Hz, which provides the basis for fast and reliable quantitation of whole lipid classes within one chromatographic run [37,38,39]. In some rare cases, molecular species of lipids are composed of exactly the same building blocks, resulting in the same elemental composition and the same fragments, which renders MRM analysis basically useless. In this case, good chromatographic separation is mandatory as shown for bis(monoacylglycero)phosphate (BMP) and cardiolipin (CL) by the group of Liebisch [38].

A good compromise between targeted and non-targeted analysis on a triple quadrupole instrument are precursor ion- and constant neutral loss scans. Although sensitivity for such scans might not be as high as in MRM mode, it opens the possibility to find unexpected species within the lipid classes surveyed by the respective precursor or constant neutral loss scans. Such systems are quite frequently used with ESI and reversed phase HPLC coupling. An excellent example of this technique is shown by Retra *et al.* [40]. The applied very shallow 60 min gradient results in baseline separation of glycerophospholipid species. The use of constant neutral loss scans for phosphatidylinositol phosphate (PIP) identification and quantitation is shown by Clark *et al.* [41].

Identification of lipid molecular species with low resolution instrumentation is best achieved by MS^n^ analysis, because this kind of analysis provides a high degree of structural information. The preferred instrumentation is either (linear) ion trap or triple quadrupole technology. Usually acquisition time for a certain compound is limited in LC-MS approaches and, as acquisition of product ion spectra takes much longer than for MRM spectra, the number of lipids to be covered is a potential bottleneck. Nevertheless, in cases of a defined number of lipids, this can be a highly specific identification strategy as shown successfully for oxysterols [42], positional isomer analysis of phospholipids [43] and in depth analysis of PIP species [44].

While chromatographic separation of lipids is often performed on reversed phase HPLC according to fatty acyl chains, this strategy runs into its limits when cholesterol esters (CE) are to be analyzed in the presence of bulk amounts of TG. Due to their very similar hydrophobicity CE and TG are hardly separated on reversed phase and hydrophilic interaction liquid chromatography (HILIC) columns, resulting in suppression of low abundant CE by TG (Figure 2). In contrast, silica-based normal phase HPLC provides separation of these lipid classes by their polar functional groups, but usually highly non polar solvents with low ionization capacity have to be used. Hutchins *et al.* [45] use APCI and post-column addition of a polar solvent to increase ionization properties of the non polar solvent eluting from normal phase HPLC. This results in a practicable online bridging between normal phase HPLC and triple quadrupole mass spectrometry, which can either be used in precursor ion, MRM or single quadrupole mode for determination of neutral lipids [34,46].

### 3.2. High Resolution Mass Spectrometry

Quadrupole-TOF mass spectrometry offers several advantages. On one hand, this instrumentation provides resolution of up to 40,000 and mass accuracy of better than 5 ppm, which is sufficient for pinning down many of the elemental compositions encountered in lipidomic analysis. On the other hand, TOF analyzers have a very high scan rate and acquire full product ion spectra very fast and efficient. On the downside is the usually limited dynamic range of the detector, which limits quantitation to a rather narrow concentration range. Nevertheless this kind of instrumentation is a valuable tool when coupled to reversed phase HPLC. Successful application of this experimental setup was used for analysis of TG and oxidized TG species. In this case, it was even possible to determine the actual fatty acid composition of TG molecular species by product ion spectra on all major species [47]. An excellent example for an integrated lipidomic platform relying on reversed phase ultra performance liquid chromatography (UPLC) quadrupole-TOF is shown by the group of Oresic [48], whereby a combination of retention time, exact precursor mass and product ion spectra are used for identification of lipids from various lipid classes. In contrast to widely used gradient elution, the group of Wenk present a profiling method based on quadrupole-TOF and isocratic reversed phase HPLC [49] used for determination of anionic glycerophospholipids, glycolipids, fatty acids, prenols and sphingolipids.

**Figure 3:**
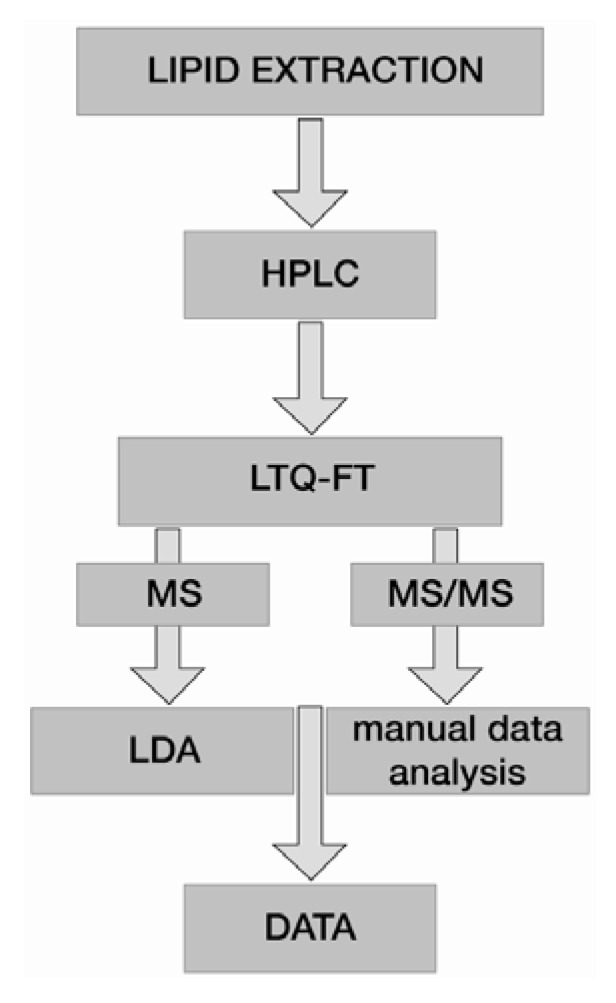
Workflow of a high resolution LC-MS platform relying on an LTQ-FT mass spectrometer. Full scan MS and MS/MS data are acquired parallel in data dependent acquisition mode. Full scan MS data are automatically processed and quantified by Lipid Data Analyzer (LDA), whereas MS/MS data are manually inspected for fatty acid assignment.

Fourier transform ion cyclotron resonance mass spectrometry (FT-ICR-MS) offers literally unlimited mass resolution and sub ppm mass accuracy. Its combination with a linear ion trap (LTQ-FT) has become a high end standard instrumentation in proteomic research, but a few groups also use it in lipidomic research. The instrument’s hybrid character holds the possibility to run the linear ion trap and the FT-ICR-MS as two instruments in parallel, resulting in high resolution precursor spectra and low resolution product ion spectra at an increased duty cycle. Coupled to HPLC, this experimental platform delivers retention time, exact sub ppm precursor masses and product ion spectra as means for identification (Figure 3). High mass accuracy paired with retention time is particularly helpful for identification of lipids with too little intensity for reliable fragment spectra. Such an integrated experimental platform is successfully used for quantitative determination of various lipid classes in lipid droplet preparations [50]. MS/MS spectra are triggered in a data-dependent manner on the four most intensive ions in the preview scan, resulting in MS/MS coverage of 66%. Owing to the ultra high resolving power and mass accuracy it is possible to confidently detect lipid species in crude lipid extracts at extremely low quantities, even when no MS/MS spectra are available for the precursor. Extension of reversed phase HPLC by a preceding HILIC fractionation of certain lipid classes results in higher sensitivity for lipid classes previously suppressed by PC. Other successful applications of similar instrumental setup are methods for quantitation of glycerophospholipids and TG in plasma samples [51] and for identification of glycerophospholipids in yeast [52]. The disadvantages of such systems are their still rather slow duty cycle of about 3 s at 200,000 mass resolution, and the low mass cutoff in the linear ion trap, which might cause loss of some low mass diagnostic fragment ions.

Within recent years, orbitrap technology has started to replace the LTQ-FT. Originally designed for small molecule identification and quantitation, this technology has a lot of advantages in store for lipidomic applications, especially when hyphenated with the fragmentation power of linear ion trap or quadrupole technology. Although resolving power and mass accuracy are less than the LTQ-FT, they are still sufficient to provide unambiguous elemental compositions for most applications. Mass accuracy can even be increased into the sub ppm range by use of constant background signals as internal lock mass calibrants. The addition of an HCD quadrupole alleviates the low mass cutoff limitations of the LTQ and provides high resolution MS/MS spectra, although at a much slower speed than for example a quadrupole-TOF analyzer. Another advantage of the most recent LTQ-Orbitrap models in comparison to the LTQ-FT is the faster scan speed of the linear ion trap at uncompromised sensitivity, allowing acquisition of up to 20 low resolution MS/MS spectra per cycle. The combination of LTQ-Orbitrap with reversed phase HPLC was successfully used by the group of Taguchi for the determination of glycerophospholipids and PIP, either by full scan of molecular ions [53] or by a combination of high resolution precursor full scans and low resolution product ion scans [53,54]. Sato *et al.* even expanded this platform by separation of glycerophospholipids into three fractions with solid phase extraction (SPE) prior to LC-MS analysis [55].

Eicosanoids are an analytical challenge because they comprise a plethora of isomeric compounds. An excellent approach by reversed phase HPLC and LTQ-Orbitrap was shown by the group of Volmer [37], whereby retention time, exact mass and MS/MS fragment intensities were all taken into account for successful identification of various isomers. A faster but somewhat less specific variant of this experimental setup is shown by the same group on an Orbitrap Exactive instrument coupled to reversed phase HPLC [56]. Although this instrumentation lacks any MS/MS capabilities, sub-ppm mass accuracies and retention time are sufficient for identification of certain glycerophospholipid classes in fast survey scans.

### 3.3. Digging Deeper into Structural Details

Beyond the actual fatty acid composition of a lipid species, the fatty acid *sn*-position can be of importance for many biological functions. Most direct infusion and LC-MS methods in lipidomics are not able to separate lipid molecular species at the level of positional isomers. However, some very sophisticated chromatographic methods are able to obtain insight on lipid molecular geometry at this level. Separation of positional TG isomers was successfully achieved by using silver-ion HPLC [57]. This publication describes a setup for serial connection of three HPLC columns to attain the necessary chromatographic resolution. Another excellent example of a highly specialized HPLC setup by the same group uses a 2D approach [58]. A first HILIC dimension separates and fractionates lipids by their respective lipid classes, and a second reversed phase dimension further separates lipid classes by their individual molecular composition. While these HPLC methods are very efficient and highly specific for separation of individual lipid molecular species, they are only suitable for a limited number of samples due to their long chromatographic separation times, particularly in 2D HPLC.

Another highly specific LC-MS strategy is proposed by the group of Blair [59]. This experimental platform couples chiral normal phase HPLC to negative APCI-MS for separation and determination of regioisomeric and enantiomeric forms of eicosanoids. Beyond enhanced separation selectivity, this method also provides very sensitive detection of eicosanoid pentafluorobenzyl derivatives by electron capture APCI and subsequent MRM analysis by triple quadrupole MS.

An important feature of molecular lipid species is the position of fatty acid double bonds. Gas phase reaction of ozone with double bonds results in primary and secondary ozonides, which fragment further to aldehydes, carboxylates and hydroperoxides indicative of the position of the double bond in the fatty acyl chain [60]. Recently, the group of Blanksby introduced custom modified instrumentation for ozone induced dissociation (OzID), at which either a linear ion trap [61] or a quadrupole collision cell [62] are able to be filled with ozone. Either sequential multistage dissociation with an inert collision induced dissociation (CID) gas and ozone, or single stage dissociation by a mixture of ozone and CID gas, results in a double bond position specific fragmentation pattern. The main limitation of this method is the specialized non-commercial equipment needed. Additionally, no high throughput standardized data analysis software is available for such an approach.

An interesting alternative for obtaining more structural details by MS/MS is the pseudo MS^3^ approach on a 4000QTrap proposed by the Merrill group [63]. Negative ESI tandem MS/MS spectra on sphingomyelin usually result in just one predominant head group specific fragment. But if the selected sphingomyelin precursor is transmitted through Q2 at low collision energies (5–10 eV) and then fragmented by the linear ion trap function in Q3, a much richer abundance of fragment ions indicative for the sphingosine backbone is to be found.

## 4. MALDI-TOF

Although not as widely used as ESI instruments, MALDI-TOF is a good complementary choice for lipids in the mass range above *m/z* 500. The soft ionization properties of MALDI result in intact molecular adduct ions. Paired with the speed of MALDI-TOF analysis this fact renders the technology very suitable for fast screening of lipids. MALDI-TOF instruments equipped with a reflectron nowadays regularly achieve 10,000 resolution and 30 ppm mass accuracy, which is sufficient for assigning intact molecular ions of lipid species. Choice of the right MALDI matrix is an important step for good sensitivity. 2,5-Dihydroxy benzoic acid, α-cyano-4-hydroxy-cinnamic acid, 9-amino-acridine and 2-mercaptobenzothiazole are often used matrix compounds. On the downside of this technology, the mass range below *m/z* 500 is usually not amenable due to matrix interferences.

MALDI-TOF has been used for analysis of various lipid classes [64], but, similar to ESI, MALDI also has certain quantitative limits for crude mixtures due to ion suppression effects [65]. This effect can become quite severe, particularly as MALDI does not allow any chromatographic separation to be coupled directly to the instrument. Recently, TLC/MALDI was proposed by several groups as an interesting alternative [66,67]. Instead of a MALDI target, a developed TLC plate with separated lipid spots is used as target. This approach is very promising, because TLC is still widely used in lipid analysis and coupling with MALDI-TOF vastly increases identification certainty of TLC spots. In such a setup, visualization of TLC spots should be reversible as well as non destructive and is either achieved by dyes non-covalently bound to lipids [67,68] or by immunodetection with antibodies [69,70]. Conventional UV lasers are used with most MALDI instruments and only penetrate the applied MALDI matrix but not the silica gel itself. Therefore UV lasers are prone to distortion of results, because only lipids at the surface of the silica particles can be detected. In contrast, IR lasers do penetrate silica gel particles and are thus certainly the better choice for TLC/MALDI targets. Although this technology is still in its infancy, there are already promising results on glycosphingolipids [69,70] and glycerophospholipids [71]. Further technological progress could develop TLC/MALDI-TOF into a fast and easy to use survey method for lipidomic analysis.

**Figure 4:**
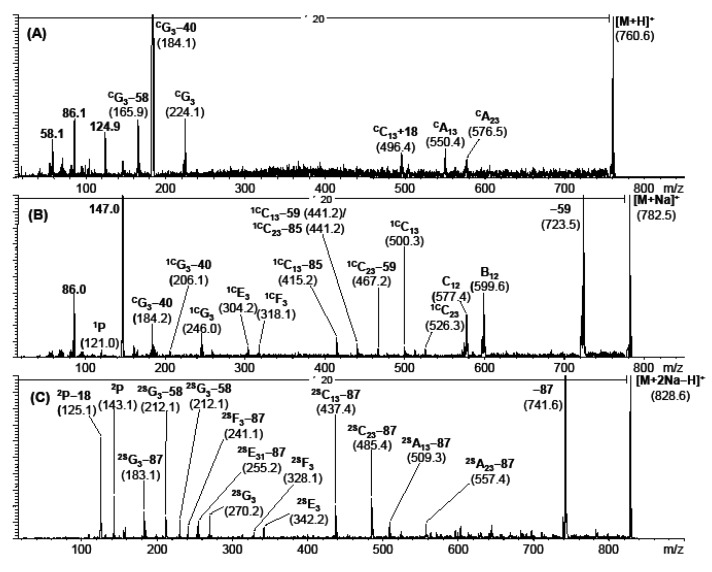
(**a**) High-energy CID-TOF/RTOF-spectrum of the [M+H]^+^ precursor ion of 1-palmitoyl-2-oleoyl-glycero-phosphatidylcholine (*m/z* 760.6); (**b**) of the corresponding [M+Na]^+^-precursor ion of 1-palmitoyl-2-oleoyl-glycerophosphytidylcholine (*m/z* 782.5), both originating from commercial hen egg lecithine; and (**c**) of the [M+2Na−H]^+^ precursor ion of 1-palmitoyl-2-arachidonoyl-glycerophosphatidylserine (synthetic origin).

While most instruments nowadays are equipped with low energy CID devices, MALDI-TOF/TOF offers an alternative for high energy CID. The advantage of high energy CID at 20 keV is induction of abundant charge remote fragmentation, which in turn allows structural analysis of lipids at the level of fatty acyl *sn* position and double bond location [72]. With the decline of sector instruments in lipid mass spectrometry, MALDI-TOF/TOF has the potential to fill this gap. Pittenauer *et al.* show the use of MALDI-TOF/TOF for such applications in an excellent manner [73,74]. Alkali cationized TG show a wealth of structure specific fragments indicative for location of fatty acids and their double bonds (Figure 4). One challenge still to be solved is the quite wide isolation window of about 4 Da which makes precursor selection especially in biological lipid extracts problematic. Although this application is clearly not a high throughput method and lacks automated software solutions, it is still highly useful for structural determination of selected lipid species.

## 5. Data Analysis

Within the last decade a constantly increasing amount of information is accessible by lipid mass spectrometry. The bottleneck in translation of acquired mass raw data into useful quantitative biological information is data processing. Data processing encompasses primarily identification and quantitation of lipid species. Inevitably, some tradeoffs like semi-quantitation instead of accurate quantitation are necessary when processing up to hundreds of lipids per analytical run. Unfortunately, no platform independent commercial lipidomic data processing software is available so far. Nevertheless, some open source software packages and one commercial vendor dependent software offer solutions for data processing. A summary about the most important features of the discussed software packages is given in Table 1.

**Table 1:** Overview about various lipidomic data processing software packages.

	Data type	MS vendor restrictions	Recommended instruments	Identification	Quanti- tation	Availability
Lipid Inspector	shotgun	all possible	triple quadrupole, quadrupole-TOF	MPIS	Yes	upon request
Lipid View	shotgun	only AB Sciex	triple quadrupole, quadrupole-TOF	MPIS	Yes	purchase
LipidXplorer	shotgun	all possible	orbitrap, quadrupole-TOF	data dependent acquisition	Yes	open source
*m/z* Mine 2	LC-MS	all possible	quadrupole-TOF	HiRes MS full scan, retention time	Yes	open source
Lipid Data Analyzer	LC-MS	all possible	FT-ICR-MS	HiRes MS full scan, retention time	Yes	open source

Developed by AB Sciex, Lipid View is the only commercially available lipidomic processing software so far. The concept of this software is based on the earlier developments of Lipid Profiler [24] and Lipid Inspector [23]. Basically all three software packages rely on shotgun data acquired by MPIS either on low resolution or high resolution instrumentation. The software processes information about precursor and fragment masses obtained from full scan and MS/MS data and matches it against an internal database containing possible fragments for molecular lipid species. On the downside, Lipid View is only able to process data acquired on AB Sciex triple quadrupole or quadrupole-TOF instruments, which severely limits the software’s availability to the lipid mass spectrometry community. 

Recently, the group of Shevchenko has launched LipidXplorer, an informatics concept based on molecular fragmentation query language (MFQL) [75]. LipidXplorer is designed for shotgun lipidomics and takes MS full scan data and product ion scans into account. Although it is also possible to process low resolution data, this program is primarily developed for high resolution spectra and was shown to work best with LTQ-Orbitrap or quadrupole-TOF instrumentation. In contrast to other shotgun lipidomics software packages, LipidXplorer does not rely on a database for MS/MS spectra but rather depends on the concept of fragmentation queries, which reflects the variability of MS/MS spectra due to different experimental settings much better. The software allows a lot of freedom for the user, like, for example, customized adjustment to various experimental parameters, but this requires some dedication.

Processing of LC-MS generated data is usually more challenging because retention time adds another dimension of information. Originally developed for metabolomic data acquired by mass spectrometry [76,77], *m/z*Mine and its sequel *m/z*Mine2 were also successfully applied on lipidomic data [78]. Following peak detection, identification of lipids is performed by searches in public libraries or customized internal databases containing exact mass and approximate retention times. Furthermore also isotopic distributions and adducts can be taken into account for lipid identification. Although originally applied on quadrupole-TOF LC-MS data, *m/z*Mine2 has become a versatile and highly flexible software, which can be used for data generated with various experimental platforms.

**Figure 5:**
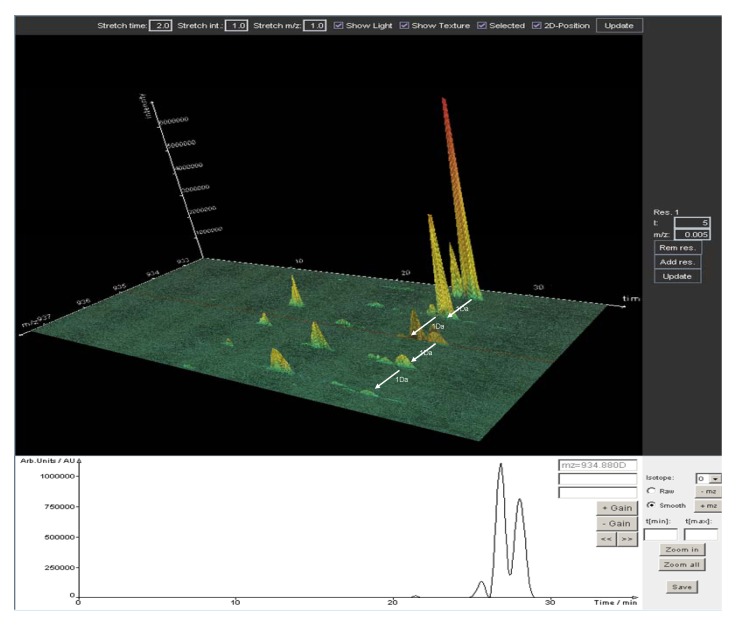
3D plot (*m/z*, retention time, intensity) of high resolution LTQ-FT data generated by Lipid Data Analyzer. Depicted are TG 56:1 and TG 56:2, including their isotopic distribution. Unambiguous identification of elemental composition is accomplished by a 3D algorithm which relies on the combination of accurate mass, retention time and isotopic pattern. The lower panel shows a 2 D plot (retention time, intensity) of *m/z* 934.88. The M + 2 peak of TG 56:2 cannot be resolved from TG 56:3 by the mass extraction window chosen, but only by retention time.

In contrast to other software solutions, Lipid Data Analyzer (LDA) is based on a 3D algorithm (*m/z*, retention time, intensity) for peak detection [79]. This offers the advantage to detect peaks with overlapping retention times and *m/z* values which might have remained undetected in a 2D plot. Identification of lipids is performed by exact mass, retention time and isotopic distribution of a compound, resulting in very high identification certainty (Figure 5). Originally designed for an FT-ICR-MS instrument, the software is highly dependent on exact mass and works best at a resolution of 100,000 or more. Nevertheless, it was also shown to perform well with quadrupole TOF data. A desirable expansion of the program would be automatic processing of MS/MS data acquired in data-dependent fashion on the most abundant *m/z* values of each high resolution full scan spectrum. Quantitation of lipids is performed with sets of internal standards covering the whole elution range of the respective lipid class. Subsequently the software performs calculations of either the mean or the median intensity of all internal standards. This procedure allows for compensation of internal standard intensity fluctuations arising from variable ion suppression effects in each elution profile.

## 6. Conclusions

Although various experimental platforms and approaches are currently established, lipidomic analysis still remains a challenge for analytical chemists and bioinformaticians alike. The biggest issue in the years to come will be standardization of data acquisition and data processing. Unlike genomic or proteomic protocols, lipidomics still stays highly diversified in instrumentation and the degree of information to be deduced from mass spectrometric data. In this respect, a standardized shorthand lipid nomenclature will be needed for database development. Furthermore, data processing is highly dependent on customized software solutions, although some promising software tools have been developed recently. Despite these challenges, it can be expected that mass spectrometry-based lipidomics will constantly develop into a high throughput technology and advance our understanding of molecular biological processes with increasing impact.
